# Genome-Wide Screen for Genes Involved in *Caenorhabditis elegans* Developmentally Timed Sleep

**DOI:** 10.1534/g3.117.300071

**Published:** 2017-07-26

**Authors:** Huiyan Huang, Chen-Tseh Zhu, Lukas L. Skuja, Dustin J. Hayden, Anne C. Hart

**Affiliations:** * Department of Neuroscience, Brown University, Providence, Rhode Island 02912; † Department of Ecology and Evolutionary Biology, Brown University, Providence, Rhode Island 02912

**Keywords:** sleep, *C. elegans*, screen, G protein, GOA-1, GPB-2, Mutant Screen Report

## Abstract

In *Caenorhabditis elegans*, Notch signaling regulates developmentally timed sleep during the transition from L4 larval stage to adulthood (L4/A) . To identify core sleep pathways and to find genes acting downstream of Notch signaling, we undertook the first genome-wide, classical genetic screen focused on *C. elegans* developmentally timed sleep. To increase screen efficiency, we first looked for mutations that suppressed inappropriate anachronistic sleep in adult *hsp*::*osm-11* animals overexpressing the Notch coligand OSM-11 after heat shock. We retained suppressor lines that also had defects in L4/A developmentally timed sleep, without heat shock overexpression of the Notch coligand. Sixteen suppressor lines with defects in developmentally timed sleep were identified. One line carried a new allele of *goa-1*; loss of GOA-1 Gα_o_ decreased *C. elegans* sleep. Another line carried a new allele of *gpb-2*, encoding a Gβ_5_ protein; Gβ_5_ proteins have not been previously implicated in sleep. In other scenarios, Gβ_5_ GPB-2 acts with regulators of G protein signaling (RGS proteins) EAT-16 and EGL-10 to terminate either EGL-30 Gα_q_ signaling or GOA-1 Gα_o_ signaling, respectively. We found that loss of Gβ_5_ GPB-2 or RGS EAT-16 decreased L4/A sleep. By contrast, EGL-10 loss had no impact. Instead, loss of RGS-1 and RGS-2 increased sleep. Combined, our results suggest that, in the context of L4/A sleep, GPB-2 predominantly acts with EAT-16 RGS to inhibit EGL-30 Gαq signaling. These results confirm the importance of G protein signaling in sleep and demonstrate that these core sleep pathways function genetically downstream of the Notch signaling events promoting sleep.

All animals sleep. The state of sleep in animals involves cessation of locomotion, species-specific posture, and increased arousal threshold with slower behavioral response to environmental stimulation (Campbell and Tobler 1984; Trojanowski and Raizen 2016). It is clear that sleep involves coordinated changes in neuronal excitability; many channel subunits and signaling pathway components have been identified as critical for sleep across species (Cirelli 2009; Sehgal and Mignot 2011; Singh *et al.* 2014; Funato *et al.* 2016). However, connections between these genes/pathways are often obscure. And, it is still unclear how the state of sleep is achieved, what mechanisms regulate the need for sleep, and how animals seamlessly transition between the sleep and wake states.

Conserved genes regulate sleep across species, including *Caenorhabditis elegans*. Numerous studies have established that conserved genes identified in other species also regulate sleep in *C. elegans*, and *vice versa* (Singh *et al.* 2014; Kucherenko *et al.* 2016; Trojanowski and Raizen 2016). *C. elegans* developmentally timed sleep occurs at the end of each larval stage during lethargus, which is coincident with shedding of the cuticle (ecdysis) (Raizen *et al.* 2008). The timing of lethargus and ecdysis is regulated, in part, by the *C. elegans* Period ortholog, LIN-42 (Monsalve *et al.* 2011). Other conserved components regulating sleep include epidermal growth factor (Van Buskirk and Sternberg 2007), Notch (Singh *et al.* 2011), protein kinase G (PKG) (Raizen *et al.* 2008), neurotransmitters (Singh *et al.* 2014), neuropeptides (Nelson *et al.* 2013; Turek *et al.* 2016), transcription factors (Turek *et al.* 2013), and G proteins (Singh *et al.* 2014; Schwarz and Bringmann 2013).

Classical forward genetic screens identify genes and pathways based on phenotypes of mutant animals. Forward genetic screens are unbiased, leverage the strengths of model organisms, and were critical in the dissection of circadian rhythm pathways (Hardin 2011), among others. We suggest that forward genetic screens will also be important for identifying critical genes and pathways required for sleep. Forward genetic screens focused on sleep have been carried out in flies and in mice (Hardin 2011; Sehgal and Mignot 2011; Funato *et al.* 2016). But, none have been focused on endogenous sleep in *C. elegans*. Thus far, identification of genes required for sleep in *C. elegans* has been achieved by testing existing mutant strains (Turek *et al.* 2013), examining candidate genes based on work in other species (Singh *et al.* 2014), or focusing only on stress-induced sleep (Iannacone *et al.* 2017).

Here, we describe the first classical forward genetic screen undertaken to identify genes regulating *C. elegans* developmentally timed sleep. We identified multiple mutagenized lines with defects in developmentally timed sleep and, thus far, have identified new alleles in two genes, *goa-1* and *gpb-2*, that are required for developmentally timed sleep. These, and additional results presented here, confirm the importance of G protein signaling pathways in sleep.

## Materials and Methods

### C. elegans culture and strain information

*C. elegans* were cultured on standard Nematode Growth Media (NGM) seeded with OP50 *Escherichia coli* and grown at 25°. Strains used in this study are shown in Table 1. Strains and alleles identified in the screen are listed in Supplemental Material, Table S2 in File S2.

**Table 1:** Strains used in the study

Strain names	Genotypes
N2	Wild Type
HA1133	*rtIs26* [*hsp*::*osm-11*;*elt-2*::*gfp*] I
HA2596	*rt167rtIs26* [*hsp*::*osm-11*;*elt-2*::*gfp*] I (2×)
HA2469	*rt186rtIs26* [*hsp*::*osm-11*;*elt-2*::*gfp*] I (2×)
DA541	*gpb-2(ad541)* I
JT603	*gpb-2(sa603)* I
CG21	*egl-30(tg26)* I; *him-5(e1490)* V
LX1226	*eat-16(tm761)* I
JT609	*eat-16(sa609)* I
MT8504	*egl-10(md176)* V
MT1443	*egl-10(n692ts)* V
MT8190	*lin-15(n765) nIs51[egl-10(+)*, *lin-15(+)]* X
LX306	*rgs-1(vs26)* III; *rgs-2(vs22)* X
LX232	*rgs-1(nr2017)* III; *rgs-2(vs17)* X

### Ethyl methanesulfonate (EMS) mutagenesis and F_2_ lines setup

L4 larvae of the strain HA1133 (*rtIs26* [*hsp*::*osm-11*;*elt-2*::*gfp*]) were mutagenized with 47 mM EMS using standard methods (Brenner 1974). Three P_0_ adult animals were allowed to lay eggs for 3 hr in a culture dish, yielding around 20–30 eggs. F_1_ generation adults were removed when ∼100 eggs were laid. Sixteen F_2_ animals were singled from each culture dish and their F_3_ generation progeny were examined in the primary screen.

### Primary screen: suppression of hsp::osm-11 adult anachronistic sleep (Ans)

Plates for F_2_ lines with adult F_3_ progeny were sealed with Parafilm (Bemis Company, Inc.) and heat shocked for 75 min, floating agar side down in a 33.5° water bath. Parafilm was removed and animals allowed to recover in a 20° incubator for 50 min. Suppression of Ans defects was scored in the next 20-min interval (between 50 and 70 min after the end of heat shock). To score Ans, plate lids were kept closed to avoid waking the animals, and plates were placed on the dissection scope agar side up, due to condensation on the inner surface of the plate lid. Only young adult animals were examined. Animals were scored as Ans if they did not move spontaneously or pump for 5 sec (Figure 2A); either pharyngeal pumping or spontaneous locomotion was sufficient to score an animal as nonAns. We retained lines with 30% or less Ans animals, examining up to 10 total young adults in each line. For lines passing this initial test, two adults were singled for rescreening in subsequent generations. If multiple nonAns lines were identified from the same P_0_, then only one nonAns line was retained after secondary screen.

### Secondary screen: Multi-Worm Tracker (MWT) assessment of L4/A sleep

The MWT assays were undertaken on plates freshly seeded with 100 μl of OP50 culture spread into a thin lawn and dried in a fume hood with the lid open for 60 min. Thirty “black dot” L4, substage L4.6 a la (Mok *et al.* 2015) L4 animals, which are about to start lethargus, were picked onto each assay plate and allowed to rest for 1 hr. Locomotion of the population was tracked for 10 min using the MWT system (Swierczek *et al.* 2011). Since the MWT requires an animal to move to start tracking, we disturbed all animals on the dish by dropping the plates 5 cm, then immediately initiated tracking. Metrics of fractional population sleep (FPS), bout frequency (BF), and mean sleep bout duration (MSBD) were calculated (see Figure 3A for definition of the metrics). The velocity of the animals was determined as reported previously (Swierczek *et al.* 2011). Sleep bouts were defined as velocity of 0 mm/sec for at least 10 sec. Metrics of FPS, BF, and MSBD were calculated with a custom Python package (CMWT, https://github.com/Huiyan-Huang/CMWT).

### Tertiary screen: microfluidic chamber-based assessment of L4/A sleep

A microfluidic, chamber-based sleep system was adapted from previous studies (Singh *et al.* 2011) and a detailed description of this protocol was published recently (Huang *et al.* 2017). Briefly, a static OP50 bacterial culture, treated with kanamycin for at least a week, was resuspended at an appropriate concentration in NGM (without agar), and applied to 6-chamber or 10-chamber microfluidic chips to facilitate loading animals and to serve as a food source. Mid-L4 stage animals, L4 substages L4.3–L4.4 (Mok *et al.* 2015), were loaded into each chamber and covered with a glass coverslip, which was then sealed to the chip using molten 2% agar. Images were recorded every 10 sec for 12 hr. Images were analyzed using a MatLab script for image subtraction (Singh *et al.* 2011) and a custom Python script for calculating lethargus duration, total sleep, and MSBD (https://github.com/Huiyan-Huang/C.-elegans-sleep-analysis, branch: Calculate-sleep-metrics) (Huang *et al.* 2017). Fractional quiescence was calculated as the rolling average over 10 min, corresponding to 60 adjacent images. The entry of lethargus (T_start_) was defined as the point at which the fractional quiescence stayed above 0.1 for at least 20 min. The exit of lethargus (T_end_) was defined as the point at which the fractional quiescence dropped to and stayed below 0.1 for at least 20 min. Total sleep is defined as the sum of time in motionless sleep bouts (10 sec and up) during lethargus. Lethargus duration is the time between the entry and exit of lethargus. MSBD is the average sleep bout length during lethargus (Figure 4A). Note that for screening purposes, lethargus was defined behaviorally based on fractional quiescence appearing at least 1 hr after mid-L4 stage. Animals were selected based on vulval morphology. Vulval eversion or ecdysis was not independently tracked.

### Backcross and whole genome sequencing

Backcrossing of mutagenized lines was initiated using males from the original unmutagenized strain, HA1133. F_1_ male progeny were crossed to unmutagenized HA1133 females generated by *fem-3 (RNAi)*. Eight cross progeny were singled and the next generation was heat shocked to induce Ans, as described above. Six nonAns young adult animals were singled from each plate (total of 48) and their progeny were retested for suppression of Ans. Four nonAns lines from different mothers were retained and used to confirm defects in endogenous developmentally timed L4/A sleep using the microfluidic chamber assay. If at least two of these 2× backcrossed lines had defects in L4/A sleep, then two independent backcrossed lines were used for whole genome resequencing. If only one backcrossed line had defective endogenous L4/A sleep, then backcrossing was reinitiated using the 2× backcrossed strain as described above, to obtain one 4× backcrossed line for genome resequencing. DNA libraries were prepared using Illumina TruSeq DNA library prep kit. Whole genome sequencing was done with Illumina HiSeq 2000 in the Genomics Facility at Brown University and sequencing data were analyzed using CloudMap on Galaxy (Minevich *et al.* 2012). We focused on identification of EMS-induced changes that might induce stop codons, small exonic deletions that might induce frame shifts, alterations in splice junctions, or missense amino acid changes. Previously described alleles from the *C. elegans* Genetics Center and other researchers were examined to determine if candidate genes were required for normal sleep during L4/A lethargus.

### Arousal threshold assessment using blue light

Arousal threshold assays were conducted as previously described (Chao *et al.* 2004) with modifications. NGM agar plates were spread with a thin 100 μl lawn of OP50, then allowed to dry overnight (lid closed) or 20 min (lid open) in a fume hood. L4/A lethargus animals at L4 substage L4.7–L4.9 (Mok *et al.* 2015) were picked onto assay plates and allowed to recover for 15 min. During the assay, the plates remained unperturbed with the lids off. A 5 mW, 405 nm laser pointer was used as stimulation, occluded with a black paper except for a small hole ∼0.5 mm in diameter in the center. A constant-current regulator set to 0.28 amperes was used to power the laser light to achieve an intensity of ∼100–110 lux. Under a dissection stereomicroscope, animals were located and stimulated with the most intense portion of the laser beam over the head of the nematode. Response latency for animals in both sleep bouts and motion bouts was recorded. For animals in sleep bouts, a response was defined as any movement. For animals in motion bouts, only forward moving animals were tested and a response was defined as the initiation of backward locomotion. Light intensity was between 95 and 120 lux and did not deviate >30 lux over the course of the experiment. At least eight animals per genotype and per condition (asleep or in motion) were tested for each trial. At least three independent trials were conducted. The experimenter was blinded as to genotype.

### Statistics

Statistical analysis was conducted using two-tailed Student’s *t*-test and *F*-test for nonzero slope for Figure S2 in File S1.

### Data availability

Strains are available upon request. Scripts used to analyze the data are uploaded to https://github.com/Huiyan-Huang. Whole genome sequencing data have been uploaded to NCBI Sequence Read Archive (#SUB2845268: SRR5811622-SRR5832). File S1 contains detailed descriptions of all supplemental files.

## Results

### Primary screen: suppression of hsp::osm-11 adult anachronistic sleep after heat shock

**Figure 1:**
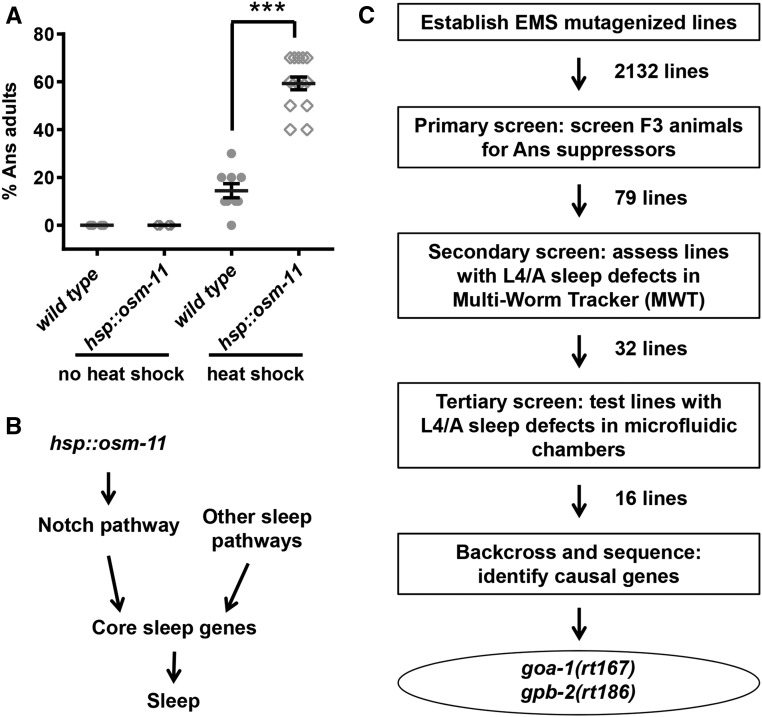
Screening rationale and strategy. (A) Heat shock-induced OSM-11 overexpression drives anachronistic sleep (Ans) at all stages, included in adult animals shown here. Each data point represents % Ans animals for an individual plate of animals. Trials were run independently on at least three different days for each genotype/treatment. *** *P* < 0.001. (B) Genetic pathways promoting sleep. OSM-11 expression activates Notch pathway signaling and, consequently, activates downstream core sleep pathways (*e.g.*, *egl-4* PKG) to promote both anachronistic sleep in adult animals and endogenous, developmentally timed L4/A sleep. Other sleep pathways (*e.g.*, *lin-42*) likely act independently to activate core sleep pathways and promote sleep. (C) Flow chart of the screen and results. Two thousand one hundred and thirty-two F_2_ lines were established and the F_3_ progeny were screened for suppression of the Ans phenotype. Seventy-nine independent Ans suppressor lines were retained and tested for defects in endogenous L4/A lethargus sleep in population-based assays using a Multi-Worm Tracker system. Detailed analysis of L4/A lethargus sleep for 32 lines was carried out in microfluidic chambers and 16 lines showed L4/A sleep defects. Seven lines were backcrossed and their genomic DNA was sequenced to find causal alleles. Genes whose loss of function results in sleep defects were identified for two mutant lines and were confirmed using previously described alleles.

**Figure 2:**
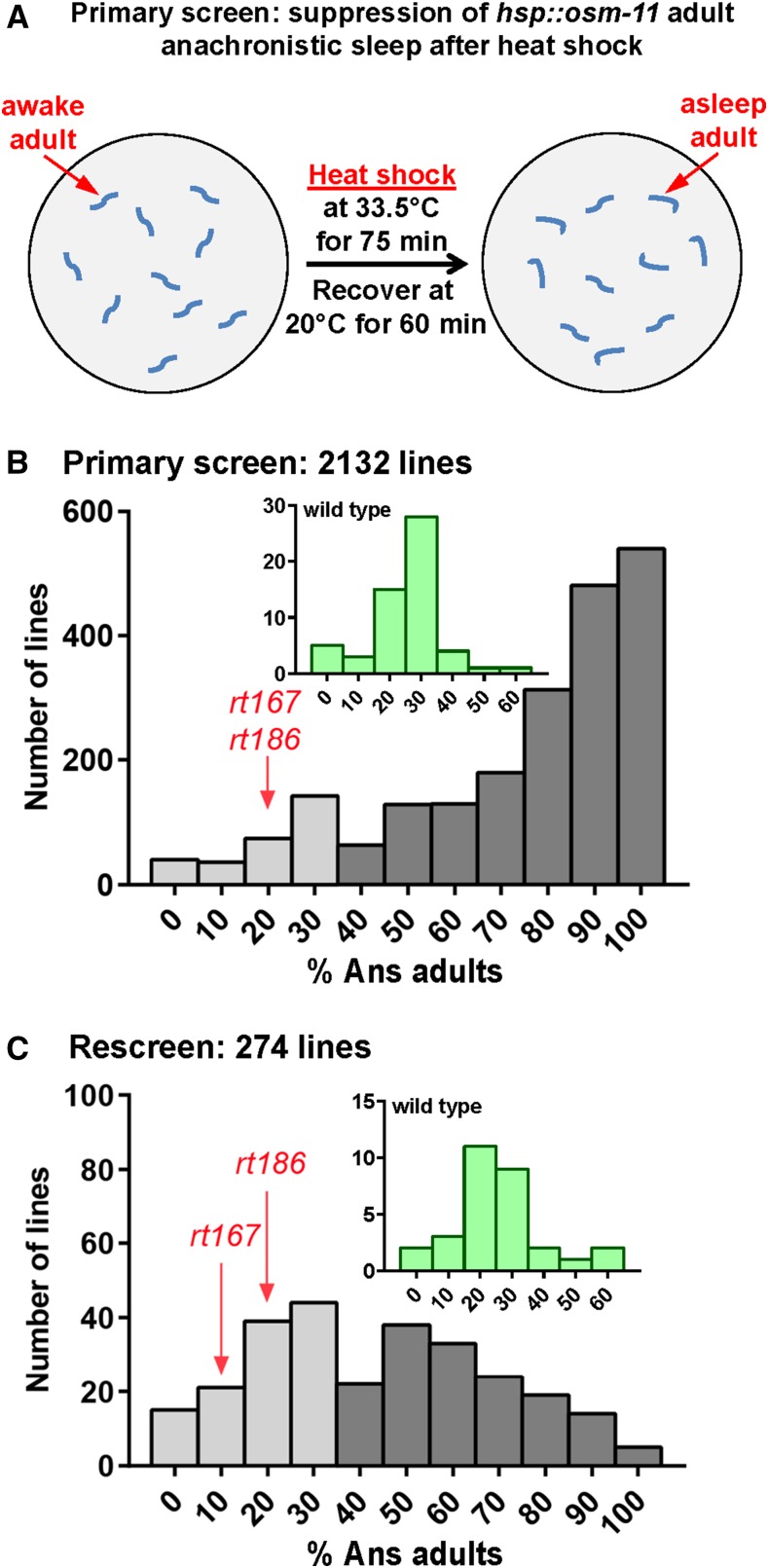
Primary screen: suppression of *hsp*::*osm-11* adult anachronistic sleep. (A) Diagram of anachronistic sleep suppression primary screen. After heat shock, suppression of Ans was assessed for each mutagenized line. (B) Histogram of primary screen results. Two thousand one hundred and thirty-two *hsp*::*osm-11* lines were tested and plates with no more than 30% Ans adults were considered candidate Ans suppressor lines. Two hundred and ninety-two lines (light gray bars) initially suppressed Ans. These were retested in the next generation for suppression of Ans. (C) 118 lines, corresponding to 79 independent F2 lines, passed the rescreen for Ans suppression. The average of two plates scored for each line is shown for the rescreen. *goa-1(rt167)* and *gpb-2(rt186)* are indicated. Plates containing heat shocked wild-type animals were used as controls during the screen; Ans observed on control animal plates is shown in insets.

To identify genes involved in sleep, we undertook a classical forward genetic screen. To facilitate identification of these genes, the screen first focused on identification of mutant alleles that suppressed the ectopic, anachronistic sleep (Ans) induced in adult *C. elegans* by transient overexpression of the Notch coligand OSM-11 (Singh *et al.* 2011). After recovery from heat shock, a large fraction of young adult *hsp*::*osm-11* animals are immobile due to Ans, compared to similarly treated wild-type animals (Figure 1A and Figure 2A). We assumed that decreased function of genes acting downstream of the Notch ligand or genes required for sleep (core sleep genes) might be identified as suppressors of Ans in a forward genetic screen (Figure 1B). Therefore, we mutagenized *hsp*::*osm-11* animals, established F_2_ lines, and screened heat shocked F_3_ adult progeny for suppression of the Ans phenotype in our primary screen (Figure 1C). Control *hsp*::*osm-11* animals showed 60% Ans under screening conditions; we retained 292 mutant lines with 30% or less Ans adults, considering these to be putative Ans suppressor lines (Figure 1B) as their fractional Ans was similar to wild-type populations (insert, Figure 2B). Next, putative Ans suppressor lines were rescreened to eliminate false positives. We randomly singled two adults from each Ans suppressor line and rescreened sublines in next generation. We were able to retest 274 putative Ans suppressor lines (Figure 2C); 118 lines showed suppression of Ans in both sublines. When multiple Ans suppressor lines were derived from the same P_0_ animal, it was possible that related lines might carry the same mutation. To avoid duplication of effort in the next stages of the screen, we randomly eliminated 38 lines and retained 79 independent Ans suppressor lines for secondary screening (Figure 1C).

### Secondary screen: Multi-Worm Tracker assessment of L4/A population sleep (no heat shock)

**Figure 3:**
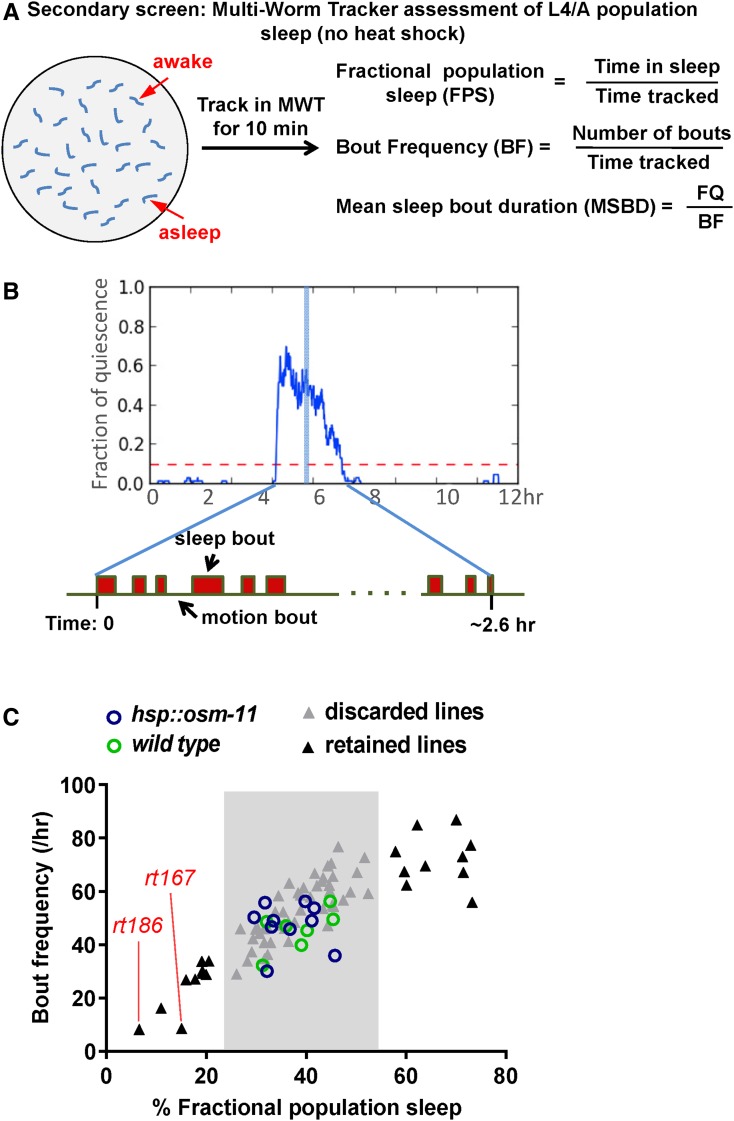
Secondary screen: Multi-Worm-Tracker (MWT) assessment of endogenous L4/A sleep. (A) Diagram of population sleep assay using MWT. A population of at least 30 midlethargus, freely roaming L4/A animals were tracked for 10 min using the MWT for each line. These animals were not heat shocked. Population sleep was measured using metrics defined here. Fractional population sleep (FPS) is the total time in sleep for all animals divided by the total time tracked for all animals. Bout frequency (BF) is the number of bouts (of at least 10 sec) for all animals divided by the total time tracked for all animals. Mean sleep bout duration (MSBD) is FPS divided by BF, which is total time in sleep divided by number of bouts. Each data point represents the mean of at least two independent trials on different days. (B) During L4/A lethargus, *C. elegans* cycles between sleep bouts and motion bouts. The illustration indicates approximate time of MWT population tracking (blue column) during L4/A lethargus. (C) Plot of BF (*y*-axis) *vs.* FPS (*x*-axis) for all Ans suppressor lines tested. Control strains are shown as circles; there is no significant difference between wild-type animals (green circles) and non-heat-shocked *hsp*::*osm-11* animals (blue circles). Ans suppressor lines are shown as triangles; lines retained for tertiary screening are indicated (outside pale gray boxes, black triangles). *goa-1(rt167)* and *gpb-2(rt186)* are indicated.

**Figure 4:**
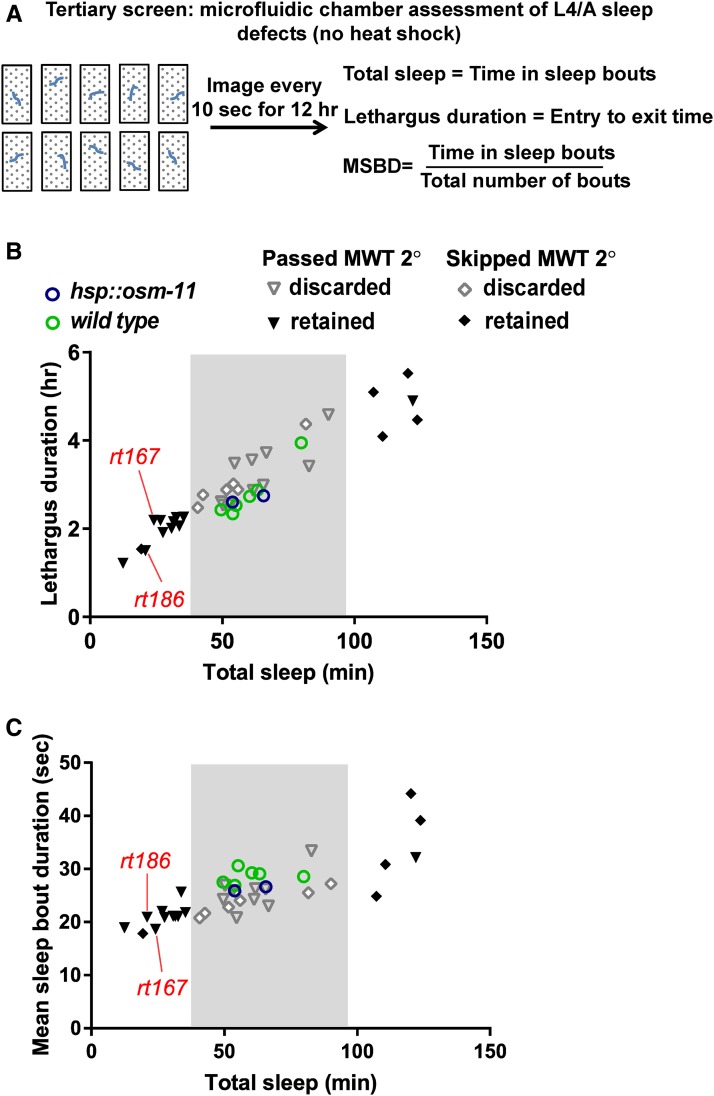
Tertiary screen: microfluidic chamber sleep assay. (A) Diagram of the microfluidic chamber assay. Early- to mid-L4 animals were loaded into microfluidic chambers and imaged every 10 sec for 12 hr (as in Figure 3B). These animals were not heat shocked. Sleep during lethargus was measured for each animal tested using the metrics defined previously and shown here. Total sleep is the sum of time in sleep bouts during L4/A lethargus. Lethargus duration is defined as the time between the appearance and cessation of frequent sleep bouts. We did not determine if vulval eversion or ecdysis timing was changed for Ans suppressor lines. Mean sleep bout duration (MSDB) is the total sleep divided by the total number of sleep bouts during lethargus. (B) Plot of lethargus duration (*y*-axis) *vs.* total time in L4/A sleep (*x*-axis) for all lines tested in the tertiary screen. (C) Plot of MSBD (*y*-axis) *vs.* total time in L4/A sleep (*x*-axis) for the same lines in B. Each data point represents the mean of at least 10 animals from each line/genotype. Control strains are shown as circles. There is no significant difference between wild-type (green circles) and *hsp*::*osm-11* (blue circles) animals. Lines that passed the preceding MWT secondary screen are shown as triangles; lines tested here that bypassed the MWT secondary screen are shown as diamonds. Sixteen lines altered L4/A lethargus sleep and were retained (outside pale gray boxes, black triangles, or black diamonds). *goa-1(rt167)* and *gpb-2(rt186)* are indicated.

This screen was undertaken to identify genes required for developmentally timed sleep. We were uninterested in mutations that suppressed OSM-11 overexpression-induced Ans due to perturbation of the heat shock pathway or mutations that decreased expression from the *hsp*::*osm-11* transgene. Additionally, an unknown fraction of Ans might be attributable to induction of stress-induced sleep (Hill *et al.* 2014). To winnow the Ans suppressor lines and identify mutant alleles that affect endogenous developmentally timed sleep, we examined L4/A sleep in the Ans suppressor lines in the absence of heat shock. Conveniently, without heat shock, the L4/A sleep of *hsp*::*osm-11* animals is indistinguishable from wild-type animals (Figure 3C and Figure 4, B and C).

To rescreen expeditiously, we developed a population-based L4/A fractional sleep assay as a secondary screen (Figure 3A). During L4/A lethargus, sleep bouts constitute roughly 30% of lethargus (Figure 3B). Mutations that decrease sleep often result in animals with less frequent, fewer or shorter sleep bouts during lethargus; conversely, mutations that increase sleep can have more frequent, more, or longer sleep bouts. We assumed that strains with dramatic differences in L4/A total sleep could be identified by sampling a population of animals for a short period of time during L4/A lethargus. We defined a new metric of “fractional population sleep” (FPS) as the fractional time a population spends in sleep bouts during the time interval sampled. However, sleep bouts are not evenly dispersed during lethargus. To maximize sensitivity, we examined previously published mutant animals and chose to measure FPS at 1 hr after lethargus entry. A previously described system that simultaneously tracks locomotion of multiple animals on culture plates, called the Multi-Worm Tracker (MWT) (Swierczek *et al.* 2011) was adapted for FPS measurement. To rescreen Ans suppressor lines for defects in L4/A lethargus sleep, we selected late-L4 stage animals based on vulval morphology and measured FPS at 1 hr into L4/A lethargus (Figure 3B). Each Ans suppressor line was tested in three independent trials with 30–40 animals each trial, sampling only 10 min of sleep for the population during L4/A lethargus.

Animals of unusual size, posture, or uncoordinated locomotion cannot be tracked accurately with the MWT system. Eleven Ans suppressor lines fell into this category and were passed to the tertiary screen below, without evaluation in the secondary screen. Population sleep was examined for the remaining 68 lines. In addition to FPS, we defined additional population sleep metrics for the secondary screen: bout frequency (BF) and mean sleep bout duration (MSBD), as shown in Figure 3A. FPS and BF were strongly correlated within each mutant line (Figure 3C); there was little variation in MSBD across mutant lines (Table S1 in File S2). We used FPS to select mutant lines for further analysis. Decreased Notch signaling can lead to either less sleep or more sleep during L4/A lethargus. Animals carrying strong loss-of-function alleles are easy to wake from sleep bouts and spend less time sleeping during lethargus; animals with less severe alleles are easy to wake, but they increase sleep quantity, presumably to compensate for poor sleep quality (Singh *et al.* 2011). Accordingly, we retained 21 Ans suppressor lines that had either decreased or increased FPS for the tertiary screen, along with the 11 Ans suppressor lines that could not be assayed in the MWT (Figure 1C and Figure 3C).

### Tertiary screen: microfluidic chamber assessment of L4/A sleep defects (no heat shock)

To confirm that mutant alleles in the remaining 32 mutant lines altered developmentally timed sleep, we used a previously established microfluidic chamber-based system (Singh *et al.* 2011) to examine endogenous L4/A sleep, without heat shock (Figure 4A). Individual late L4 animals were loaded into microfluidic chambers and images were captured every 10 sec for 12 hr, which encompasses the entire L4/A lethargus (Figure 3B). Previously described analysis programs were used to detect movement and sleep, based on image subtraction. Lethargus entry, exit, and sleep metrics were determined, including total sleep, lethargus duration, and MSBD. At least 10 animals were examined for each Ans suppressor line in the tertiary screen (see Figure 4A and *Materials and Methods* for details). Sixteen mutant lines had significant sleep defects during L4/A lethargus, when compared to wild type and *hsp:osm-11* control animals (Figure 4, B and C and Table S2 in File S2). Animals from some mutant lines sleep less than control animals; animals sleep more in other lines.

### Whole genome sequencing finds two causal genes for sleep defects: goa-1 and gpb-2

We undertook further analysis of seven mutant lines with unequivocal L4/A sleep defects. To decrease the number of nonpertinent, EMS-induced nucleotide changes, we backcrossed mutant lines to the original *hsp*::*osm-11* strains and reisolated homozygous mutant animals, based on suppression of Ans and L4/A sleep defects. For each line, genomic DNA from either one 4× backcrossed line or two independent 2× backcrossed lines was sequenced to identify candidate causal alleles. We assumed that causal alleles would be homozygous in backcrossed lines and unique to each line. After genome resequencing, roughly 10–20 homozygous, unique exonic changes were identified as candidate alleles for each mutant line. To identify causal alleles and corresponding genes, we determined if L4/A sleep was perturbed by preexisting alleles or RNAi knockdown of candidate genes. Animals expressing the SID-1 double-stranded RNA channel in neurons were used for RNAi feeding studies. Using this approach, *goa-1* and *gpb-2* were successfully identified for causal alleles *rt167* and *rt186*, respectively. The phenotypes of lines carrying *rt167* and *rt186* at each stage of the screen are indicated in Figure 2 and Figure 4. Both suppressed Ans, reduced population sleep in the MWT, and reduced total sleep in microfluidic chamber assays.

**Figure 5:**
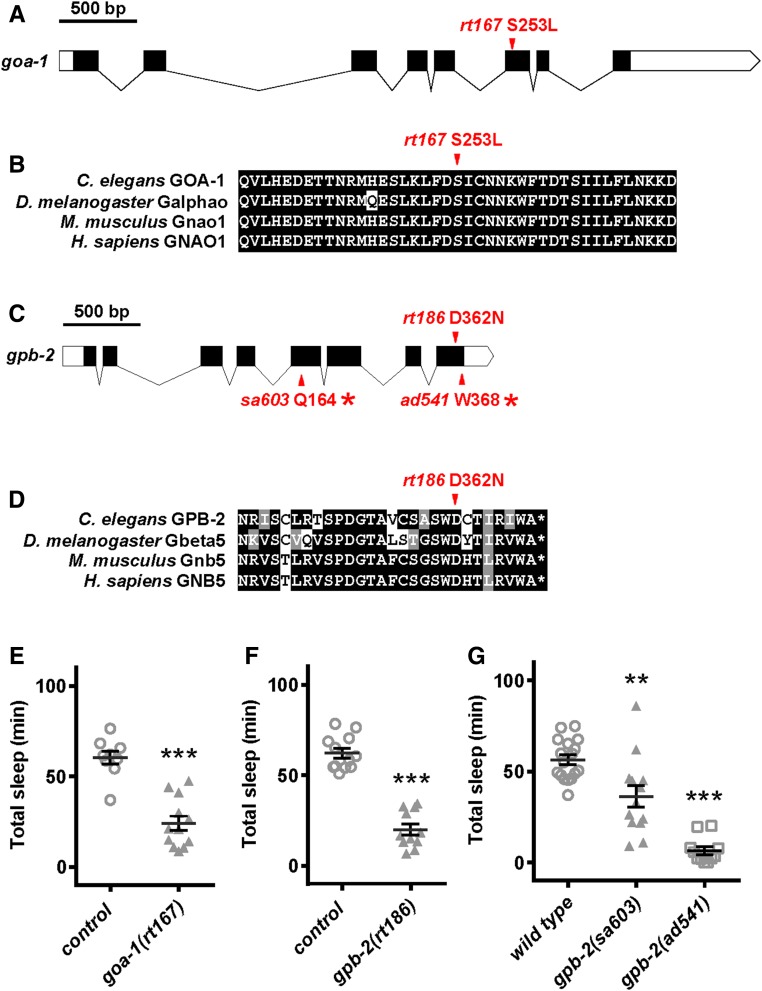
Identification of *goa-1* and *gpb-2* alleles. (A) Diagram showing location of *goa-1(rt167)* in the sixth exon of *goa-1*. (B) Alignment of amino acids adjacent to *goa-1(rt167)* showing cross-species conservation. (C) Diagram showing location of *gpb-2(rt186)* in the last exon of *gpb-2*, as well as preexisting loss-of-function alleles that were examined herein. (D) Alignment of amino acids adjacent to *gpb-2(rt186)* showing cross-species conservation. (E) *goa-1(rt167)* animals had reduced total sleep compared to control *hsp*::*osm-11* animals. None of these animals were heat shocked. Results from 2× backcrossed animals shown. (F) *gpb-2(rt186)* animals had reduced total sleep compared with control *hsp*::*osm-11* animals. None of these animals were heat shocked. Results from 2× backcrossed animals shown. (G) Animals carrying preexisting *gpb-2* alleles had decreased total sleep, compared with wild-type animals. Mean and SEM indicated, along with results for individual animals for each genotype. ** *P* < 0.01, *** *P* < 0.001 by Student’s *t*-test *vs.* control or wild-type animals run in parallel in multiple trials.

*rt167* is a missense mutation in *goa-1*, converting serine 253 to leucine (S253L). Serine 253 is highly conserved across animal species (Figure 5, A, B, and E) (Slep *et al.* 2008). Previous studies have established that loss of Gα_o_ GOA-1 function leads to decreased sleep in animals (Guo *et al.* 2011; Singh *et al.* 2014; Schwarz and Bringmann 2013); isolation of *goa-1(rt167)* confirms the utility of screening strategies outlined here. *gpb-2(rt186)* is a missense mutation converting a highly conserved aspartic acid at position 362 to asparagine (D362N) in the Gβ_5_ protein GPB-2 (Figure 5, C and D) (Cheever *et al.* 2008). We ascribed sleep defects to loss of *gpb-2* function as animals carrying reduced function alleles *gpb-2(sa603)* and *gpb-2(ad541)* (Robatzek *et al.* 2001) had decreased L4/A sleep (Figure 5G). However, we realized that due to the unique function of GPB-2, biased alleles might be a potential issue (Porter *et al.* 2010 and see below). We also determined that *gpb-2* loss-of-function animals had reduced arousal thresholds during L4/A sleep bouts (Figure S1 in File S1), consistent with defects in sleep. Therefore, the first genome-wide, forward genetic screen for genes involved in developmentally timed sleep revealed a previously unknown role for GPB-2 in sleep. Identifying two proteins involved in G protein signaling, despite the relatively low number of mutagenized lines screened, highlights the importance of this pathway and warranted further investigation.

### RGS proteins EAT-16, RGS-1, and RGS-2 are required for normal L4/A sleep

In *C. elegans*, previous work suggested that GPB-2 directly binds to and is required for the function of two regulator of G protein signaling (RGS) proteins: EAT-16 and EGL-10 (van der Linden *et al.* 2001). GPB-2 helps these RGS proteins activate the intrinsic GTPase activity of Gαq EGL-30 and Gα_o_ GOA-1, respectively (Chase *et al.* 2001; Robatzek *et al.* 2001). These Gα proteins play antagonistic roles in synaptic release regulating locomotion and egg-laying. Since both EGL-30 and GOA-1 have been previously demonstrated to play antagonistic roles in *C. elegans* sleep (Schwarz and Bringmann 2013; Singh *et al.* 2014), it was not surprising that GPB-2 loss also impacted *C. elegans* sleep. However, considering the antagonistic roles of GOA-1 and EGL-30, loss of GPB-2 function theoretically could have resulted in no overall net impact on sleep or resulted in poorly regulated sleep, with highly variable sleep quantity. We consistently observed reduced sleep in *gpb-2* loss-of-function animals, suggesting a biased impact of G protein signaling on L4/A lethargus sleep or a biased selection of alleles tested (Porter *et al.* 2010). Mutations in GPB-2 could have two distinct effects: some reducing or eliminating all GBP-2 functions, and others preferentially inactivating GPB-2/EAT-16 complex while leaving GPB-2/EGL-10 function relatively intact (Porter *et al.* 2010). Based on egg-laying and locomotion phenotypes of the two *gpb-2* alleles tested here, sa607 reduces GPB-2 function while *ad541* is biased toward inactivating GPB-2/EAT-16 complex. To circumvent the potential allele bias of *gpb-2*, we decided to determine the function of EAT-16 and EGL-10 in sleep, respectively.

**Figure 6:**
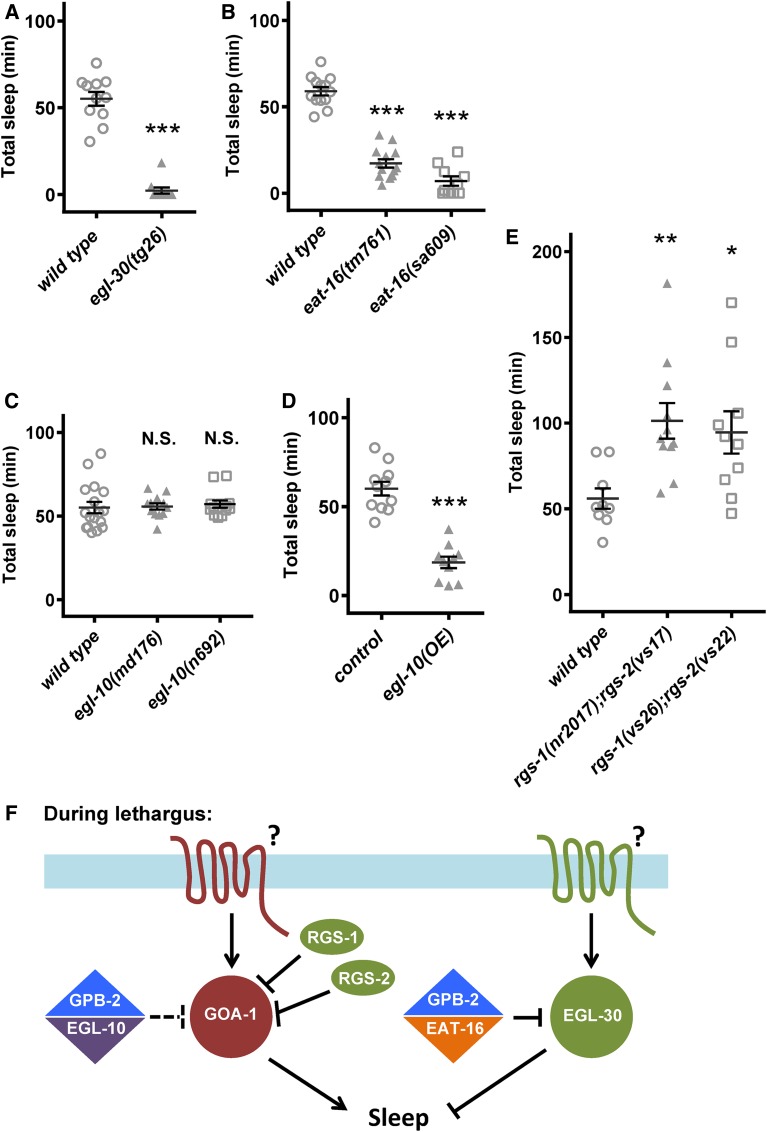
GPB-2 and RGS-1/2 regulate Gα_q_ EGL-30 and Gα_o_ GOA-1, respectively, in L4/A lethargus sleep. (A) *egl-30*(*tg26*) gain-of-function animals had dramatically decreased total sleep during L4/A lethargus in microfluidic chamber assays, consistent with a previous report. Severe or complete loss of *egl-30* causes paralysis or lethality. (B) Loss of function in the RGS protein EAT-16 led to decreased L4/A lethargus sleep, consistent with EAT-16 loss leading to increased EGL-30 Gα_q_ activity. (C) Loss of RGS protein EGL-10 function had no impact on L4/A lethargus sleep, suggesting that other RGS protein(s) regulate pertinent Gα_o_ GOA-1 activity. Only L4/A total sleep is shown here, but lethargus duration and sleep bouts duration were also not affected (data not shown). (D) Increasing EGL-10 RGS protein activity, using a previously described overexpression strain, was sufficient to decrease L4/A lethargus sleep. (E) Simultaneous loss of both RGS-1 and RGS-2 (RGS-1/2) led to increased sleep. Two previously described double mutant strains were examined. Increased sleep observed here is consistent with RGS-1/2 loss leading to increased GOA-1 Gα_o_ activity (due to decreased GTP hydrolysis), which would cause more sleep. Mean and SEM indicated, along with results for individual animals for each genotype. * *P* < 0.05, ** *P* < 0.01, *** *P* < 0.001 by Student’s *t*-test *vs.* control or wild-type animals run in parallel in multiple trials. (F) G protein pathway signaling pertinent to L4/A lethargus sleep. On the right side of the illustration, Gβ_5_ GPB-2 and RGS EAT-16 proteins normally act together to increase EGL-30 Gα_q_ GTP hydrolysis, which decreases overall signaling via EGL-30. EGL-30 Gα_q_ is normally wake-promoting; increased EGL-30 or gain of function drives less sleep. The identity of putative G protein receptor(s) that drive EGL-30 signaling is unclear. On the left side of the illustration, loss of GOA-1 Gα_o_ decreases sleep, suggesting that GOA-1 Gα_o_ and effectors are normally sleep-promoting. In other behavioral contexts, Gβ_5_ GPB-2 and RGS EGL-10 proteins act together to regulate GOA-1 Gα_o_ activity, but loss of EGL-10 had no impact on L4/A lethargus sleep. However, loss of both RGS-1 and RGS-2 function altered sleep suggesting that at least one of these RGS proteins acts to increase GOA-1 Gα_o_ GTP hydrolysis, which would decrease overall signaling via GOA-1 and downstream effectors. The GPCR receptors that drive increased GOA-1 Gα_o_ activity are also unknown. The balance of the Gα_q_ and Gα_o_ signaling is a major factor in determining the quantity of sleep seen in L4/A lethargus.

To first confirm the role of *egl-30* in sleep, we examined animals carrying *egl-30* gain-of-function alleles (Figure 6A) and verified reduced L4/A sleep (Figure 6A). Consistent with this observation, two loss-of-function alleles of *eat-16* also resulted in decreased sleep, consistent with previously described actions of these proteins (Figure 6B). Unexpectedly, two independent loss-of-function alleles of *egl-10* did not lead to L4/A sleep defects in either total sleep, lethargus duration, or mean sleep bout duration, despite our finding that overexpression of EGL-10 in multi-copy arrays was sufficient to decrease sleep (Figure 6, C and D). These two results suggest that *egl-10* is normally dispensable in this paradigm; alternative RGS proteins may normally be required to regulate GOA-1 function during L4/A lethargus and permit sleep. Likely candidates for these alternative RGS proteins were RGS-1 and/or RGS-2. These two proteins act redundantly to inhibit GOA-1 signaling and allow reinitiation of egg-laying after refeeding post starvation (Dong *et al.* 2000). We found that animals lacking both *rgs-1* and *rgs-2* had increased sleep, which is consistent with their loss leading to a presumable increase in GOA-1 signaling (Figure 6E). We conclude that, during lethargus, GPB-2 mainly interacts with EAT-16 to attenuate EGL-30 signaling, while GOA-1 signaling is negatively regulated by RGS-1 and RGS-2. It remains unclear if EGL-10 plays a role in endogenous lethargus sleep, but EGL-30 Gα_q_ signaling and GOA-1 Gα_o_ signaling are critical for normal L4/A sleep, and their function is dependent on RGS-1, RGS-2, EAT-16, and GPB-2 (Figure 6F).

## Discussion

Here we describe the first classical, forward genetic screen for genes involved in *C. elegans* developmentally timed sleep. We identified one gene previously implicated in sleep (*goa-1*) and one gene previously not implicated (*gpb-2*). Examination of loss-of-function alleles for G protein signaling pathway components revealed that GPB-2 likely acts with EAT-16 to inhibit EGL-30 Gαq signaling in this paradigm. Moreover, examination of other RGS proteins revealed that GOA-1 Gαo signaling is regulated by RGS-1 and RGS-2 during L4/A lethargus, rather than EGL-10.

Starting with 2132 mutagenized F_2_ lines, we used primary, secondary, and tertiary screening strategies to identify 16 mutant lines with defects in endogenous, developmentally timed L4/A sleep. Reflecting back on our design of the screen, we appreciate the convenience of basing the primary screen on suppression of ectopic anachronistic sleep (Ans). This approach significantly reduced the number of lines examined in secondary and tertiary screens for endogenous L4/A sleep defects. We acknowledge that relying on suppression of Ans in the primary screen means that only genes downstream of Notch signaling and/or core sleep genes can be identified.

Having completed the screen, we reexamined the effectiveness of the population-based MWT assay as a screening strategy to identify strains with developmentally timed sleep defects. One measure of effectiveness is the success rate in tertiary screening for lines passed from the MWT secondary screen *vs.* lines that skipped the MWT. Of the 21 lines that had population sleep defects in the MWT, only 11 showed sleep defects in the microfluidic chamber assay: roughly a 50% success rate. Of the 11 lines that bypassed MWT secondary screening due to size, posture, and/or locomotion defects, five had sleep defects: again, roughly a 50% success rate. Two-sided Fisher’s exact test suggests that there was no obvious benefit from MWT (*P* > 0.9999). But, this comparison might be flawed as locomotion defects that cause tracking problems in MWT might also affect sleep assessment. However, we do not generally find that locomotion defects preclude accurate sleep assessments in microfluidic chamber assays. Therefore, as an alternative strategy to retrospectively assess the utility of the MWT assay for sleep studies, we examined the correlation between MWT results and microfluidic chamber results for each mutant line. We plotted MWT FPS *vs.* microfluidic chamber total sleep; no significant correlation was seen (*R*^2^ = 0.026, *P* = 0.48, Figure S2 in File S1). Admittedly, it is possible that causal alleles were not homozygous during secondary screening, but this seems an uncommon scenario. Overall, we do not recommend the MWT population-based assays to assess L4/A sleep defects. Instead, we strongly recommend using the established microfluidic chamber assay, which we used here for tertiary screening.

How many *C. elegans* genes are important for L4/A lethargus sleep? An earlier study estimated that roughly 15% of *Drosophila* genes affected sleep (Harbison and Sehgal 2008). Here, we started with 2132 F2 mutagenized lines and yielded 16 mutant lines with endogenous sleep defects. For an F2 recessive screen of this design (50 mM EMS and sparse screening of F2 plates), standard calculations suggest that a homozygous loss-of-function allele for an average *C. elegans* gene will be found once for every 5000 F2 lines examined (Brenner 1974; Greenwald and Horvitz 1980; Shaham 2007). Based on our results, one might suggest that the *C. elegans* genome has roughly 38 genes that both suppress Ans and dramatically perturb L4/A lethargus sleep, which would include Notch downstream target genes and core sleep genes. This is likely a dramatic underestimate for several reasons. First, we note that the ineffective secondary screen using the FPS/MWT likely led us to discard many mutant lines carrying alleles that impact developmentally timed sleep, perhaps up to 50%. Considering this, the genome likely contains >80 genes required for Ans whose loss also dramatically alters endogenous sleep. Next, we note that our screening strategy focused on identifying alleles that cause dramatic sleep defects; genes with more subtle defects or changes in lethargus timing would not be identified. Finally, we suggest that not all genes involved in sleep will be required for Ans, which is induced by Notch pathway activation. Based on these considerations, we conclude that a rational estimate of the number of genes required for sleep in *C. elegans* cannot yet be made.

Here, we identified mutant strains that suppressed anachronistic, adult sleep (Ans) in adult animals. Some suppressor strains decreased total L4/A sleep, which seems counterintuitive. We suggest three possible explanations. First, some strains may carry two pertinent mutations in two different genes; one mutation that only decreases Ans sleep and one that fortuitously increases L4/A sleep. Although many genes are required for sleep, this should be a rare occurrence. Second, a single mutation in a suppressor strain may decrease Ans sleep and increase L4/A sleep. But genes whose perturbation simultaneously leads to both of these defects have not been reported previously. Third, Ans strains may carry mutations in genes whose perturbation makes sleep less restorative, leading to compensatory increases in sleep quantity. Strong decrements in Notch pathway signaling lead to decreased L4/A sleep and inappropriately low arousal thresholds only during L4/A sleep bouts. (These animals sleep less and are easy to wake.) But, milder decrements in Notch pathway signaling lead to **increased** L4/A sleep and inappropriately low arousal thresholds only during L4/A sleep bouts. A similar suite of defects was seen in *C. elegans* with decreased Jnk pathway signaling (Singh *et al.* 2011). It was suggested that inappropriate low arousal thresholds signify poor sleep quality, which engages homeostatic pathways and results in compensatory increases in sleep quantity. Given that (i) a third of the mutant strains had increased sleep, (ii) this suite of defects has been observed for Notch pathway genes, and (iii) the primary screen relied on suppression of a Notch pathway gain-of-function defect, we conclude that Ans lines with increased L4/A sleep likely carry mutations in Notch pathway genes or transcriptional targets.

The first genes we identified in this screen were *goa-1* and *gpb-2*, both G proteins. G protein pathways are well conserved and play diverse roles in signaling events across species. Overexpression of the *Drosophila* GOA-1 ortholog induces sleep, while decreasing Gα_o_ signaling by RNAi leads to fragmented sleep (Guo *et al.* 2011). However, to our knowledge, Gα_q_ signaling has not been previously implicated in sleep in species other than *C. elegans* (Schwarz and Bringmann 2013), while Gβ_5_ has not been implicated in sleep at all. Gβ_5_ knockout mice are developmentally impaired and have multiple neurologic abnormalities including hyperactivity, which might lead to reduced sleep (Zhang *et al.* 2011). We suggest that the Gα_q_ signaling and Gβ_5_ activity are also required for normal sleep in other animal species.

RGS proteins are important modulators of the G protein signaling and are expressed at high levels in neuronal tissues (Ross and Wilkie 2000; Neubig and Siderovski 2002). There are >20 genes encoding RGS proteins in mammals and 13 genes in *C. elegans*. RGS proteins accelerate the slow intrinsic hydrolysis rate of GTP to GDP by Gα proteins. A recent study examined RGS-insensitive Gα_i2_ knock-in mice, finding that RGS and Gα_i2_ protein activity modulates wakefulness, NREM sleep, and REM sleep (Zhang *et al.* 2016). Although no specific RGS proteins were tested in this study, other reports suggest that RGS proteins are involved in sleep and circadian rhythm regulation. The expression of RGS16 is circadian and is critical for the time-dependent activation of intracellular cyclic AMP signaling in the suprachiasmatic nucleus (SCN), a critical circadian center regulating behavioral rhythms (Doi *et al.* 2011). There is also evidence that RGS4 and RGS2 protein levels respond to melatonin signaling; protein levels peak during the middle of the night and decline to basal levels during the day (Dupre *et al.* 2011; Matsuo *et al.* 2013). A large-scale human GWAS study identified a locus near RGS16, an established circadian gene, as associated with self-reported early wakefulness (Hu *et al.* 2016). Moreover, tumor necrosis factor may induce central nervous system dysfunction, including lethargy, by up-regulation of RGS7 (Benzing *et al.* 1999). EGL-10 and EAT-16 are the *C. elegans* orthologs of RGS7, and human Gβ_5_ specifically binds RGS6, RGS7, RGS9, and RGS11 (Witherow and Slepak 2004). Additional studies will be required to determine if RGS7 loss alters sleep in mammals by modulating Gα_o_ or Gα_q_ signaling.

Classic genetic screens provide an unbiased strategy for identifying genes playing unsuspected roles in any biological process, if robust screening strategies can be established. Given the proven utility of this approach for other behaviors and our relatively poor understanding of the mechanisms underlying sleep despite decades of effort, we suggest that classical forward genetic screens will provide novel insights into the conserved genes and pathways important for sleep across the animal kingdom.

## Supplementary Material

Supplemental material is available online at www.g3journal.org/lookup/suppl/doi:10.1534/g3.117.300071/-/DC1.
