# Supplementary material for: Genome-Wide Screen for Genes Involved in Caenorhabditis elegans Developmentally Timed Sleep
Source: G3 (Bethesda). 2017 Jul 26;7(9):2907–17. doi: 10.1534/g3.117.300071 (PMC5592919; doi:10.1534/g3.117.300071)
Supplement: Supplementary file 1 [file 2907FileS1.pdf]

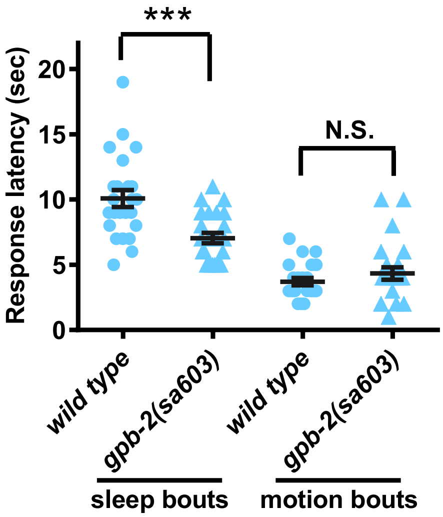

Figure S1. *gpb-2* loss of function animals have decreased arousal thresholds only during sleep bouts. Response latency (time to respond in seconds, y-axis) to the noxious stimulus of intense blue light was decreased in *gpb-2(sa603)* loss of function animals during sleep bouts, compared to wild type animals. During L4/A lethargus motion bouts, animals lacking *gpb-2* respond as swiftly as control animals to the same stimulus, ruling out a pervasive sensory response defect. At least 8 animals per trial for each genotype/condition were tested in at least 3 independent trials. ***: $p<0.001$ by Student's T-test, with mean and SEM indicated.

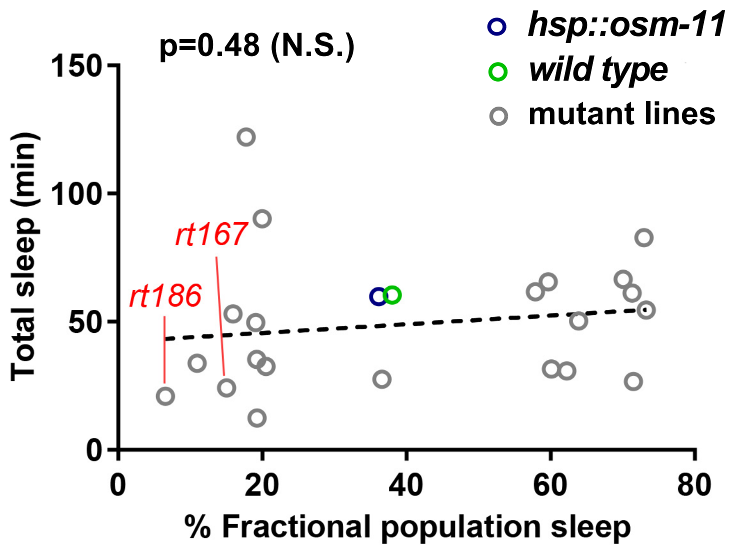

Figure S2. The correlation of the MWT assay and the microfluidic chamber assay. We examined the correlation in assessments for these two assay systems for each mutant line tested in both assays (n=21). The average of results for all wild type animals (green circle) and non-heat shocked *hsp::osm-11* animals (blue circle) is indicated. Results for *goa-1(rt167)* and *gpb-2(rt186)* are also indicated. Mean total sleep from microfluidic chambers assays (y-axis) is graphed *versus* FPS (fractional population sleep) from MWT assay (x-axis). No correlation was observed. Because the microfluidic chamber assay is likely more accurate, we do not recommend using MWT assay to assessing L4/A lethargus sleep in future studies or screens. Using the MWT as a secondary assay likely led us to erroneously discard Ans suppressor lines containing alleles that perturb L4/A lethargus sleep. The data points were fitted into a linear equation of Y=0.1698*X+42.24 (black dotted line) yielding R^2 = 0.02646 and p = 0.4812, indicating no significant correlation.

Supplemental Tables (see excel file)

Supplemental Table 1. Secondary screen: lines retained based on MWT results. Results are shown as mean ± SEM for all trials and metrics; MWT output for each trial is available upon request. Lines carrying *goa-1* and *gpb-1* alleles are indicated in red. n.c.: inconclusive results due to insufficient time tracked. n.d.: not determined due to obvious locomotion defects.

Supplemental Table 2. Tertiary screen: microfluidic chamber results. All lines tested in microfluidic chambers during the tertiary screen are reported in this file; individual trial results are available upon request. Only one representative control line is included. Controls were interspersed periodically to confirm calibration the microfluidic chamber assay systems. To determine significance for mutant lines, the most recent flanking control determination were used. Results are shown as mean ± SEM. Alleles of identified genes were indicated in red. Mutant lines with strong L4/A lethargus sleep defects are shaded in light grey. *: $p<0.05$ in Student's T-test used for the decision to retain the line as a mutant line with altered sleep.

Supplemental Table 3. List of sample names on Sequence Read Archive (#SUB2845268) and their corresponding strain names, genotypes, and SRA accession #.

Supplemental Table 4-14. List of homozygous mutations affecting coding regions in each strain sequenced. Each table is named with their strain name.
